# Autoimmune primary adrenal insufficiency -current diagnostic approaches and future perspectives

**DOI:** 10.3389/fendo.2023.1285901

**Published:** 2023-11-10

**Authors:** Anette S. B. Wolff, Isil Kucuka, Bergithe E. Oftedal

**Affiliations:** ^1^ Department of Clinical Science, University of Bergen, Bergen, Norway; ^2^ Department of Medicine, Haukeland University Hospital, Bergen, Norway

**Keywords:** primary adrenal insufficiency (PAI), glucococorticoids, 21-hydroxylase autoantibodies, Addisons disease, Genetic causes of PAI

## Abstract

The adrenal glands are small endocrine glands located on top of each kidney, producing hormones regulating important functions in our body like metabolism and stress. There are several underlying causes for adrenal insufficiency, where an autoimmune attack by the immune system is the most common cause. A number of genes are known to confer early onset adrenal disease in monogenic inheritance patterns, usually genetic encoding enzymes of adrenal steroidogenesis. Autoimmune primary adrenal insufficiency is usually a polygenic disease where our information recently has increased due to genome association studies. In this review, we go through the physiology of the adrenals before explaining the different reasons for adrenal insufficiency with a particular focus on autoimmune primary adrenal insufficiency. We will give a clinical overview including diagnosis and current treatment, before giving an overview of the genetic causes including monogenetic reasons for adrenal insufficiency and the polygenic background and inheritance pattern in autoimmune adrenal insufficiency. We will then look at the autoimmune mechanisms underlying autoimmune adrenal insufficiency and how autoantibodies are important for diagnosis. We end with a discussion on how to move the field forward emphasizing on the clinical workup, early identification, and potential targeted treatment of autoimmune PAI.

## 1 Adrenal insufficiency

**Figure 1:**
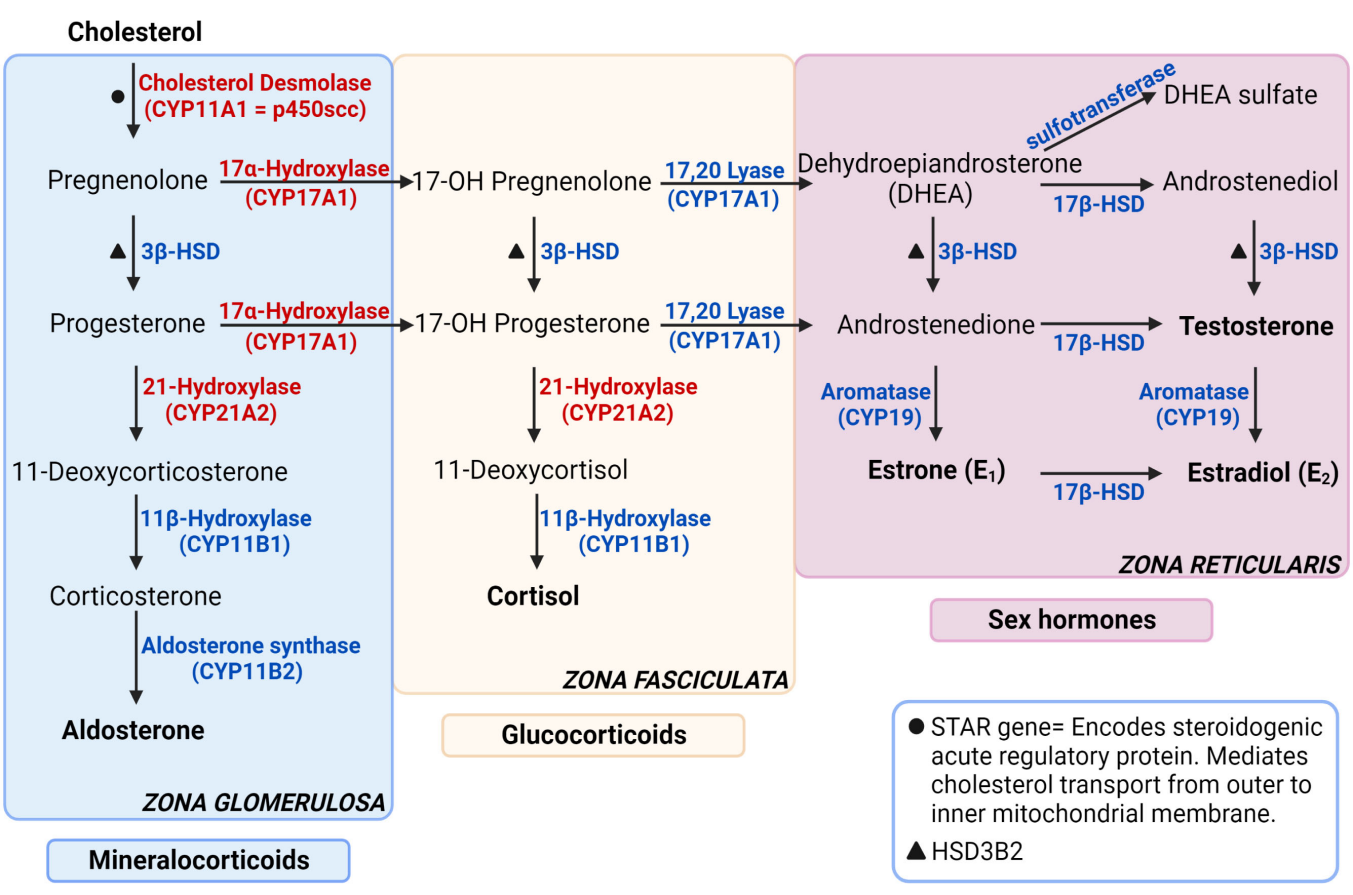
Steroidogenesis pathway. The enzymes specified are encoded by the genes stated in parenthesis under respective enzymes. The genes that are not stated in parenthesis are represented with symbols. The enzymes in red are targets for autoantibodies in autoimmune primary adrenal insufficiency. Figure created in BioRender.com. Modified from (1).

Adrenal glands regulate functions such as metabolism, blood pressure and the stress response by producing hormones from the two main parts it consists of: cortex and medulla. The adrenal cortex is responsible for producing glucocorticoids, mineralocorticoids, and adrenal androgens whereas the medulla produces adrenaline and noradrenaline, also referred to as catecholamines (**Figure 1**). Glucocorticoids, primarily cortisol, also known as the stress hormone, have numerous effects on metabolism. It increases gluconeogenesis in liver, elevates blood sugar levels by inhibiting glucose intake into the cells and by suppressing insulin secretion, regulates the body’s stress response, increases blood pressure and heart rate, and suppresses inflammation (1). Mineralocorticoids, primarily aldosterone, maintain salt-water balance and regulate blood pressure by increasing sodium and water reabsorption into circulation and potassium excretion from kidneys. Adrenal androgens are dehydroepiandrosterone (DHEA), dehydroepiandrosterone sulfate (DHEAS) and androstenedione.

**Figure 2:**
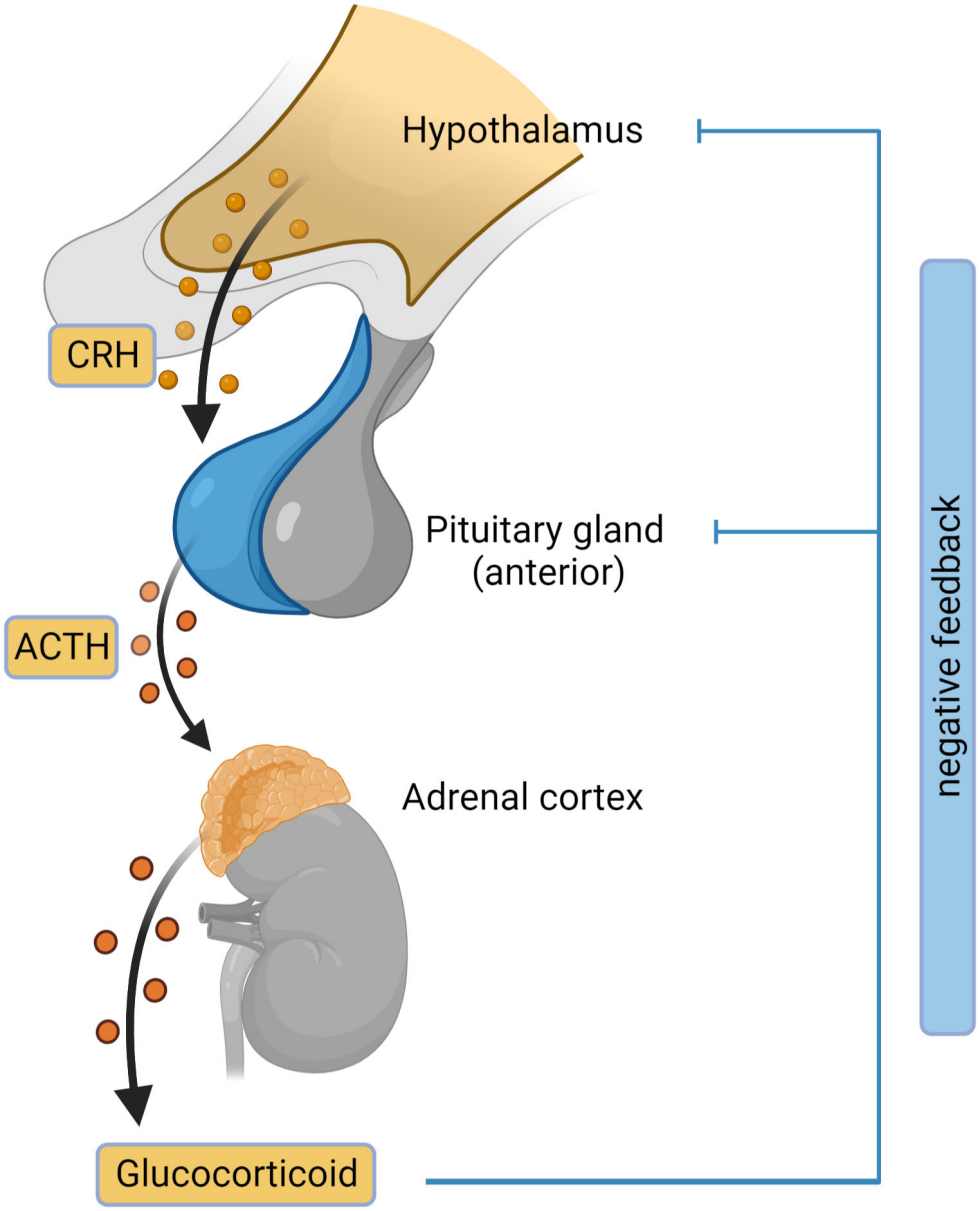
Hypothalamic-pituitary-adrenal (HPA) axis. The outline of the HPA axis between the hypothalamus, pituitary gland and the adrenal cortex is shown, including the negative feedback-loop. CRH, corticotropin-releasing hormone; ACTH, adrenocorticotropic hormone. Figure created in BioRender.com.

Adrenal insufficiency (AI) is the condition where the glucocorticoids are not produced adequately because of a decreased function of adrenal glands (2). There are 3 main types of adrenal insufficiency: primary (adrenal), secondary (pituitary) and tertiary (hypothalamic). Tertiary AI is often incorporated in Secondary AI category without additional distinction. Primary AI (PAI), also known as Addison’s disease, is a rare disease with a prevalence of approximately 100 per million in Europe (3–5). PAI is caused by damage or malfunction of the adrenal glands, resulting in insufficient production of cortisol, aldosterone, and DHEA. In secondary adrenal insufficiency, inadequate adrenocorticotropic hormone (ACTH) levels cause reduced stimulation of adrenal glands leading to decreased glucocorticoid secretion. Adrenal insufficiency caused by exogenous steroid treatment is a common type of AI. Exogenous glucocorticoid treatment suppresses the hypothalamic-pituitary-adrenal axis by negative feedback mechanisms, leading to low corticotropin-releasing hormone (CRH), ACTH and inadequate cortisol production (**Figure 2**) (6, 7).

The most common cause of primary adrenal insufficiency is autoimmunity; this type is often called Autoimmune Addison’s Disease, or simply autoimmune PAI. Other causes include, but are not limited to, congenital adrenal hyperplasia, surgical removal of adrenal glands (bilateral adrenalectomy), damaged adrenal glands as a result of infection or hemorrhage and more recently, as an adverse event of cancer immune checkpoint inhibitor therapy (8) (**Table 1**). Autoimmune PAI can appear as an isolated manifestation but is in >60% of cases seen concomitantly with other autoimmune disorders in autoimmune polyglandular syndromes (APS) (9). A recent study comprising > 22 million persons in the United Kingdom revealed an increased risk of autoimmune PAI connected with almost any other autoimmune disease, either linked to joint genetic risk factors, or the commonly used treatment for several autoimmune disorders with high doses of glucocorticoids (10).

**Table 1:** Causes of primary adrenal insufficiency.

Aetiology	Example
**Autoimmunity**	Autoimmune primary adrenal insufficiency Autoimmune polyendocrine syndrome type-2 Autoimmune polyendocrine syndrome type-1
**Infection**	Tuberculosis (*Mycobacteria*) Septic shock, Meningococcal sepsis Bacteria (*Neisseria meningitidis, Haemophilus influenzae, Pseudomonas aeruginosa*) Virus (*HIV, Herpes Simplex Virus, Cytomegalovirus*) Fungi (*Pneumocystis jirovecii*)
**Tumour**	Primary tumours Metastatic cancers Adrenal lymphoma
**Infiltration**	Amyloidosis Haemochromatosis Sarcoidosis Histiocytosis
**Adrenal Hemorrhage**	Anti-phospholipid syndrome Disseminated intravascular coagulation Anticoagulant therapy
**Trauma/Surgery**	Tumour surgery Bilateral adrenalectomy (e.g. Cushing’s syndrome) Radical nephrectomy
**Genetic**	Congenital adrenal hyperplasia Congenital lipoid adrenal hyperplasia Adrenoleukodystrophy (X-linked) Kearns-Sayre Syndrome Smith-Lemli-Opitz Syndrome ACTH resistance (familial glucocorticoid deficiency type 1) (familial glucocorticoid deficiency type 2)
**Adrenal Dysgenesis**	Adrenal hypoplasia congenita
**Medication**	Ketoconazole, Fluconazole, Etomidate, Mitotane, Aminoglutethimide, Metyrapone, Rifampin, Phenytoin, Phenobarbital, Mifepristone.

## 2 Clinical overview

Clinical manifestations of PAI can be grouped depending on the deficient hormones. Glucocorticoid deficiency related symptoms are muscle and joint pain, weakness, anemia, loss of appetite, weight loss, and low blood pressure. Maintenance of blood glucose concentration is damaged, causing hypoglycemia, exhaustion, and fatigue. In addition, patients may present with slightly elevated levels of the thyroid stimulating hormone (TSH). Hypercalcemia can also be seen in PAI, but it is uncommon (2, 11). Hypercalcaemia is presumably due to increased bone resorption caused by raised TSH commonly seen in PAI due to the lack of the inhibitory effect of glucocorticoids on pituitary TSH secretion (12). Symptoms related to low Aldosterone levels include hyponatremia, hyperkalemia, salt craving and abdominal pain. Serum electrolyte disturbances lead to dizziness, nausea, vomiting and low blood pressure. Symptoms like low energy, reduced sexual responsiveness, lack of libido in women, and erectile disfunction in men are associated with deficiency of adrenal androgens. Other symptoms of PAI include dry skin and patchy hyperpigmentation in oral mucosa and areas with increased friction such as palmar creases, axillary region, and dorsal foot.

### 2.1 Diagnosis

Suspicion of adrenal insufficiency through clinical manifestations is the first step to diagnosis. Clinical presentation of patients is often non-specific, which may lead to delayed diagnosis. Common symptoms and findings include weight loss, fatigue, postural hypotension, and hyperpigmentation; the latter stands out as notably specific to primary adrenal insufficiency. In routine laboratory assessments, hyponatremia, hyperkalemia, and hypoglycemia increase clinical suspicion of PAI. Next step for suspected PAI is assessing the adrenocortical function through the diagnostic test: paired measurement of serum cortisol and plasma ACTH. Morning cortisol concentration <140 nmol/L and an ACTH concentration twice the upper reference limit verifies a PAI diagnosis (2).

In ambiguous cases with morning cortisol >140nmol/L, cosyntropin stimulation test (also known as ACTH 1-24 stimulation test or short synacthen test) is performed. Cosyntropin, a synthetic form of ACTH, stimulates secretion of adrenocortical hormones from the adrenal glands. The principle of cosyntropin stimulation test is to measure plasma cortisol concentrations before and after injecting cosyntropin. A healthy person is expected to have >500 nmol/L of plasma cortisol concentration 60 minutes after 250µg cosyntropin injection. The first blood sample taken before the cosyntropin injection is to measure the baseline plasma cortisol levels. After injection, at 30 and 60 minutes, blood samples are taken for cortisol assessment. The threshold in this test at 60 min. is 500 nmol/L, defining cortisol concentrations <500 nmol/L as adrenal insufficiency (13). Low-dose (1µg) ACTH stimulation test has also been proposed as an alternative diagnostic test for adrenal insufficiency. Although the low-dose test is closer to physiological ACTH secretion levels in comparison to 250µg stimulation test, studies have shown that it is not superior in diagnostic accuracy to the standard test (14, 15). Low-dose test is prone to be affected by technical difficulties of the process such as dilution of the preparation, precise measurement, and injection (15). Even small volume loses can lead to significant differences in the ACTH dose delivered and affect the results. Because of these differences, although the low-dose test can also be used in clinical practices, 250 µg ACTH stimulation test remains as the gold standard test and is recommended. In addition to cortisol and ACTH, plasma renin and aldosterone levels are assessed. Low aldosterone and high renin concentrations (or high plasma renin activity) indicate mineralocorticoid deficiency (2).

After a PAI diagnosis is made, further investigation starts to determine the cause of adrenal failure. In Western countries, where other than autoimmune causes of PAI are uncommon, serum 21-hydroxylase antibodies should be assessed. Positive autoantibody evaluation confirms the diagnosis as autoimmune primary adrenal insufficiency (autoimmune PAI) and requires screening for autoimmune comorbidities such as autoimmune thyroid disease, coeliac disease, and type 1 diabetes. The monogenic APS-1should be considered in patients under 20 years old (6, 16). In patients with negative autoantibody test, the next step is CT imaging of the adrenal region, which could diagnose infections, hemorrhage, or infiltrations such as tumors in the area. In male patients with negative 21-hydroxylase autoantibody tests, very long chain fatty acid (VLCFA) levels should be measured in serum to eliminate adrenoleukodystrophy (2, 13). Patients who present with suspected adrenal crisis should start treatment as quickly as possible, without being delayed by diagnostic assessments.

### 2.2 Treatment

The principle of adrenal insufficiency treatment is replacement of deficient hormones (**Table 2**). The main goals of the treatment are to improve the life quality of patients, reaching optimum glucocorticoid regimen, avoiding comorbidities caused by glucocorticoid over-replacement such as metabolic syndrome, and preventing mortality associated with adrenal crisis. In PAI, patients need replacement of both glucocorticoids and mineralocorticoids (2, 17). The standard medication of glucocorticoid treatment for patients with AI (primary and secondary) is hydrocortisone or cortisone acetate (6, 18, 19). Patients with primary AI additionally take fludrocortisone treatment for mineralocorticoid deficiency. Unrestricted sodium intake and avoiding salt craving are also parts of the mineralocorticoid substitution therapy. These patients are allowed to use salt without restrictions and recommended to consume salty food as needed (11).

**Table 2:** Standard substitution doses of glucocorticoid and mineralocorticoid treatment in adrenal insufficiency.

Hormone replacement therapy	Treatment
Hydrocortisone*	TabletsAdults (18 years or older) : 10-25 mg daily dose (in 2-4 doses) ** **Examples: 10mg + 5mg + 5mg, ** 7.5mg + 5mg + 2.5mg,** ** 10mg + 5mg + 2.5mg,** ** 10mg + 10mg,** ** 10mg + 5mg + 5mg,** ** 10mg + 5mg + 5mg + 5mg** Children-Adolescents (up to 18): 8-10 mg/m^2^ (in 3-4 doses, 50-66% in morning dose) Modified release Adults (18 years or older) : 15-25 mg once daily Capsules Children-Adolescents (up to 18): 8-10 mg/m^2^ (in 3-4 doses, 50-66% in morning dose)
Fludrocortisone**	(*Only given in primary adrenal insufficiency*)*!* Adults (18 years or older) : 0.05-0.20 mg once daily Children with 6-17 years of age: 0.075-0.100 mg/m^2^ once daily Children with 1-12 years of age: 0.100-0.150 mg/m^2^ once daily Infants (up to 2 years of age): 0.150 mg/m^2^ once daily

*Hydrocortisone for glucocorticoid substitution, **Fludrocortisone for mineralocorticoid substitution. Treatment of adrenal crisis is not included in the table.

Adrenal androgen replacement is not a part of the standard treatment, even though it is deficient in adrenal insufficiency along with glucocorticoids and mineralocorticoids. Female patients taking optimized glucocorticoid and mineralocorticoid replacement may still present with persistent low energy and/or lack of libido. In these cases, adrenal androgen replacement can be considered. The positive effects of DHEA treatment have shown to be minor; but subjective health status, mood and libido were found to be improved with treatment doses between 10mg- 25mg daily (6). Due to DHEA being converted to estrogen after intake, it poses a risk of several estrogen associated diseases such as venous embolism, cardiovascular disease, and estrogen-sensitive cancers. The risk is currently unassessed and long-term safety data are lacking, and DHEA replacement is not recommended for routine use in a recent guideline by the Endocrine Society (6, 20).

## 3 Genetics in adrenal diseases

**Figure 3:**
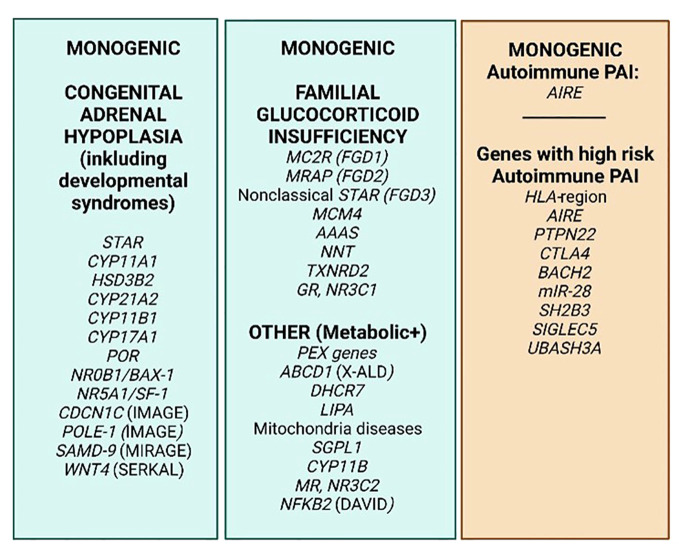
PAI genetics. The most common monogenic genetic causes of PAI (green) and 8 genes/gene regions where specific variants cause extra risk for autoimmune PAI in a polygenic setting on a genome–wide basis (orange). Based on http://www.icped.org/, and (25, 26).

With the Human Genome Project and the emerge of whole exome or genome sequencing and vast amounts of genome association studies (GWAS) that are available and affordable, prediction of disease is not only based on gender, age, known family history and biochemical patterns and markers anymore, but potentiate prediction of disease in more unbiased ways (21). A number of genes are known to confer early onset adrenal disease in monogenic inheritance patterns, including genetic defects in enzymes of adrenal steroidogenesis (e.g., *CYP21A1* and *CYP11A1*), the ACTH receptor (*MC2R*) and molecules involved in establishing the mitochondrial redox potential like nicotinamide nucleotide transhydrogenase (*NNT*) and thioredoxin reductase 2 (*TRXR2*) amongst others. Yet other genes include steroidogenic acute regulatory protein (*StAR*), *NR5A1*/steroidogenic factor-1, and *NR0B1* (DAX-1) (22–24). **Figure 3** summarizes some of the genetic defects that lead to congenital adrenal hyperplasia.

The etiological basis of the most common form of adrenal insufficiency in the western world, the autoimmune, has been linked to genetic variation in addition to unknown environmental factors (27). Skov and collaborators recently showed, by analyzing 29 twin pairs with PAI, a concordance for monozygotic twins of 0.71 and a heritability of 0.97, revealing that PAI is a highly genetic disorder (28). Informative syndromes caused by errors in essential immune genes like *AIRE* (autoimmune polyendocrine syndrome type 1, APS-1) and *NFKB2* (DAVID syndrome) causing autoimmune disease in the adrenals either as a primary or secondary, further throw light on parts of the pathogenesis of autoimmune PAI. Interestingly, rare mutations in several other immune genes have also recently been found to be likely causes for an autoimmune pathology of the adrenals, e.g., *RAG1, TNFAIP3, LAT* and *IKZF2* (29). Numerous candidate studies performed in the pre-genomic era further pointed to “PAI-associated genes”, i.e. polymorphisms that are significantly more (un)common in patients than in healthy controls, revealing several “known autoimmunity genes” to also confer risk for PAI, including *HLA, CTLA4, BACH2* and *PTPN22* (30–34).

### 3.1 Genome wide association study for autoimmune PAI

With a few exceptions, autoimmune PAI does not follow a Mendelian inheritance pattern, and is considered a complex genetic disorder. However, twin studies (28) and observation of familial clustering (3, 35) confirm the high heritability of PAI, prompting further studies. We recently performed the world’s first GWAS on autoimmune PAI including only ~1200 patients, but which succeeded to elucidate nine genetic regions with quite remarkable risk potential for PAI (21) (**Figure 3**). The success criteria relied on carefully phenotyped patients, high quality DNA and stringent criteria for inclusion, i.e., positivity for autoantibodies against one of the key enzymes for adrenal hormone synthesis, 21-hydroxylase (21OH). Indeed, the elucidated genes were predicted to cause 40 percent of the genetic risk traits for autoimmune PAI with the absolutely highest risk depending on the HLA-region (36). Several of the risk genes revealed were previously confirmed to confer risk for PAI, e.g., *BACH2, PTPN22* and *CTLA4.* Other genes were newly identified, including *LPP, BACH2, SH2B3, SIGLEC5, UBASH3A*, and intriguingly two coding SNPs in *AIRE*. Hence *AIRE* is a risk gene both in a monogenic and polygenic setting for PAI and was also recently discovered as conferring risk towards two other organ specific autoimmune disorders, namely type 1 diabetes, and pernicious anemia (37, 38). Indeed, our research shows that dosage of AIRE probably has an impact on the development of autoimmune disease (35, 39).

### 3.2 Interpreting and expanding the genetic knowledge in PAI

So how can we use this information? Eriksson and colleagues estimated with a linear model that the odds ratio for PAI more than doubled with every additional PAI risk allele when analyzing 479 Swedish PAI patients and found the subject’s age at disease onset to depend on their combined risk allele load. On average, subjects with more than nine risk alleles acquired PAI more than eight years earlier than subjects with less than five risk alleles, but no single loci could be associated to age of onset (40). Based on the risk alleles revealed in the GWAS, we established a polygenic risk score (PRS) for autoimmune PAI, revealing an average score for patients more than 1,5 SD higher than healthy controls (41). The PRS was tested in 18 pediatric patients and identified three cases of monogenic PAI which had clearly low PAI PRS-scores compared to the autoimmune patients (41). Hence, this technique might be applied to point to patients with a likely monogenic, rather than polygenic, cause of adrenal failure; i.e. direct who should be screened for mutations based on next generation sequencing efforts. The PRS can furthermore be applied to cohorts at special risk for autoimmune adrenal failure (e.g., registries of other endocrine autoimmune conditions or families with high burden of endocrine autoimmunity, or cancer patients who receive immune checkpoint therapy), providing likelihood-measures for development of PAI.

## 4 Autoantibodies in autoimmune PAI

Due to their deficiency in proper immunological tolerance mechanisms, B cells and plasma cells from PAI patients typically produce autoantibodies directed against intracellular enzymes in the affected organs. These organ-specific autoantibodies are excellent markers for autoimmune disease in the organ which they are expressed. Since autoantibodies often precede clinical symptoms, they are assayed at a routine basis (42–44).

### 4.1 Autoantibodies in PAI

Patients with pathogenic *AIRE* mutations are found to have high levels of circulating autoantibodies against cytokines, in particular interferon (IFN) omega (45–47) and interleukin (IL-)22 (48, 49). Neutralizing antibodies against IFN alpha and omega have also been detected in a number of monogenic immunodeficiencies (50–54). In a study by Sjøgren et al., 675 PAI patients were screened for autoantibodies against IFN-omega and IL-22, detecting 29 positive patients; 4 new APS-1 cases and 11 variants in immune genes in 8 patients suggesting these autoantibodies to be present in rare, genetic causes of PAI and endocrine autoimmunity (29).

More commonly, organ-specific autoantibodies are detected in PAI. The adrenal, steroidogenic P450 superfamily autoantigens 21-OH, 17-a-hydroxylase (17-OH) and side-chain cleavage enzyme (SCC) catalyze chemical reactions required for production of steroid hormones like aldosterone and cortisol (21-OH), progesterone (17-OH) and pregnenolone (SCC). Autoantibodies against SCC is a valid marker for primary ovarian insufficiency (POI), defined as menopause before 40 years of age, within the PAI cohort. POI is more commonly found among women with PAI than in non-autoimmune subjects, where 10.2% of women were diagnosed with POI in a Norwegian PAI cohort (55). SCC had the highest accuracy detecting POI, and the accuracy did not increase compared to combining the results from SCC and 17-OH autoantibody testing, suggesting that young PAI women should be routinely tested for autoantibodies against SCC as a predictive marker for POI.

21-OH is a steroid enzyme exclusively located in the adrenal cortex. It is a microsomal cytochrome P450 enzyme with two main functions; converting 17-hydroxyprogesterone to 11-deoxycortisol and progesterone to deoxycorticosterone. Like other microsomal P450s, the enzyme accepts electrons from a NADPH-dependent cytochrome P450 reductase, thus reducing molecular oxygen and hydroxylating the substrate. With its central role in the adrenal, autoantibodies targeting 21-OH is a hallmark of autoimmune PAI.

### 4.2 Autoantibodies targeting 21-OH and their diagnostic and prognostic value in autoimmune PAI

**Figure 4:**
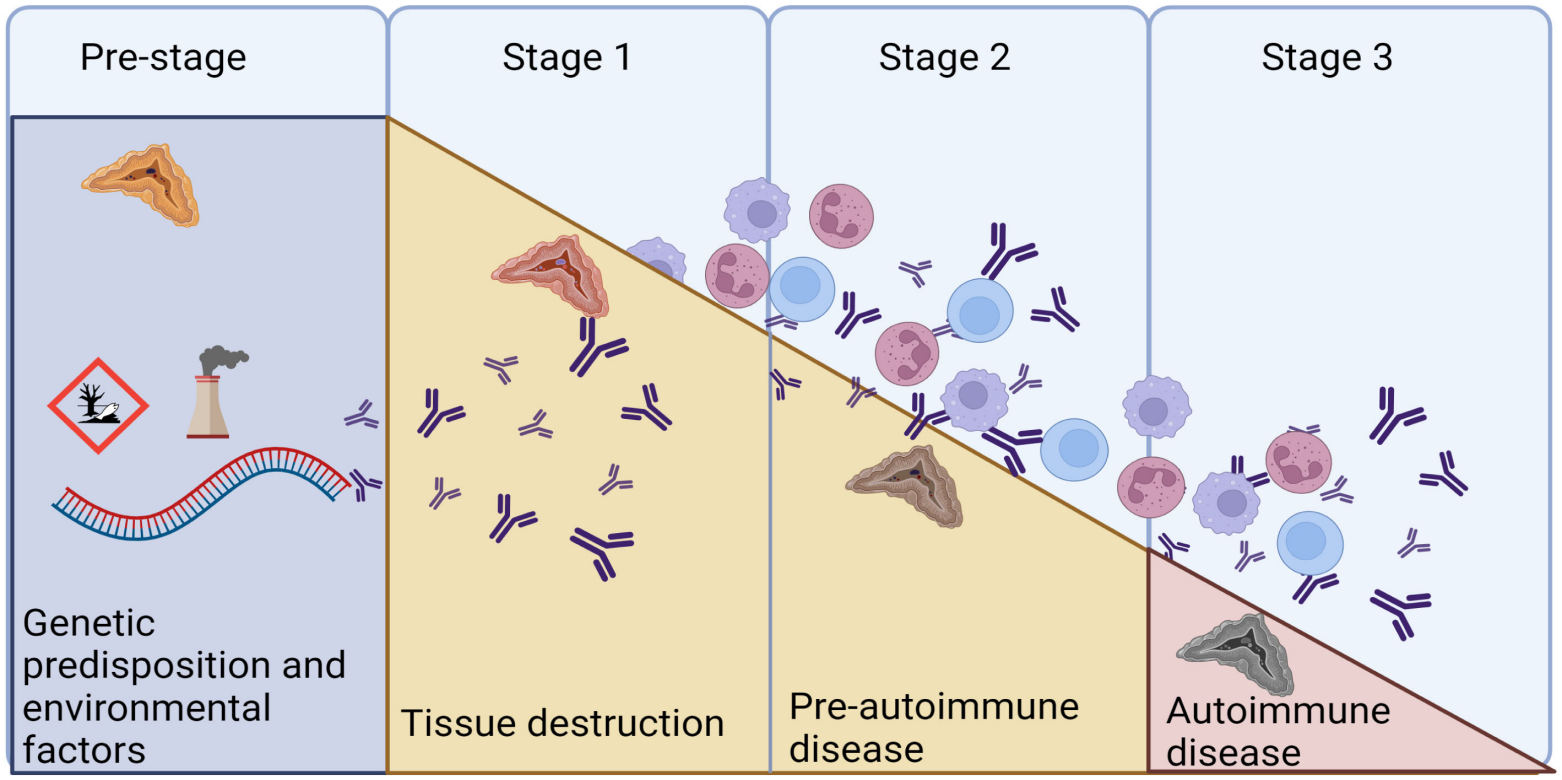
The natural cause of progression of autoimmune diseases. We are today posed to identify the genetic predisposition in AAD, and to provide replacement therapy in Stage 3, when overt disease is evident. Autoantibodies against 21OH can appear as early as in the pre-stage, as markers of the autoimmune reaction. Modified from (66) and created in BioRender.com.

The autoimmune nature of PAI is supported by the presence of autoantibodies recognizing 21-OH (21OH-Abs), found in most of the patients at diagnosis (56). The autoantibodies targeting 21-OH was first described in 1992 by Winqvist et al., identified as a major autoantigen in autoimmune PAI (57). These antibodies have since then been shown to be highly specific for autoimmune PAI in several patient cohorts (44, 58–61), and found present in 80–90% of patients with PAI in cross-sectional studies after exclusion of known non-autoimmune causes (62, 63). Hence, it is a reliable marker used in the diagnostic work up of PAI (14). Conversely, 21OH-Abs can also be found in individuals with normal adrenal function, where their presence hints to a future risk of developing overt PAI (64, 65). However, it has been shown that pre-clinical adult patients with 21-OH Abs only have a cumulative risk of about 20% of developing overt PAI if adrenal function is normal at the start of the observation (64), although these studies were clearly unpowered. However, combined with the genetic risk factors, these autoantibodies can be used to identify future PAI patients in the early stages of the disease (**Figure 4**).

Data from a small patient cohort indicates that the frequency of anti-21OH is higher shortly after diagnosis (>95%) (67), and it has been reported to decrease with increasing disease duration reaching about 50% positivity after 20 years (67, 68). We have previously investigated the levels of 21-OH Abs in a longitudinal fashion and found them to be remarkedly stable, where >90% of the patients being positive 30 years after diagnosis (61). Even though the levels of 21-OH Abs declined with increasing disease duration, it only rarely reached negativity. Importantly, a negative 21-OH Abs result does not exclude autoimmunity if assayed many years after disease onset. We found the 21-OH Abs indices to be affected by factors like age at diagnosis, sex, type of Addison’s disease (isolated vs autoimmune polyendocrine syndrome type I or II) and mostly the risk HLA genotype in the patients. These autoantibodies are also useful markers within cohorts with increased risk of developing PAI, like newly diagnosed APS-1 patients, where it is an excellent marker for predicting PAI (69, 70).

## 5 Autoimmune reaction towards adrenal gland and 21-OH

Identification of the main autoantigen (as determined by binding of autoantibodies) also points to mechanisms behind the autoimmune destruction of the adrenal glands. But as 21-OH is an intracellular enzyme, it is unlikely that the 21-OH Abs directly mediate their destruction, and they are probably rather a sign of ongoing tissue destruction and ongoing antigen presentation (71). Several lines of study support this notion. Firstly, during pregnancy a mother with PAI will transfer 21-OH Abs to the child but the disease is not transmitted (72). In line with this, histological studies of adrenal glands from deceased Addison’s disease patients show significant mononuclear cell infiltration into the adrenal gland (73). An ongoing immune reaction was further shown to be likely by Freeman et al. three decades ago, using Peripheral Blood Mononuclear Cells (PBMCs). PBMCs from PAI patients proliferated in response to adrenal proteins while control PBMCs did not (74). Also using PBMCs, Bratland et al. found that their proliferation and production of interferon-gamma in response to 21OH was significantly higher in patients compared to healthy controls. Furthermore, the 21OH-specific production of interferon-gamma was enhanced in the presence of 21OH autoantibodies. They also showed mature dendritic cells to be superior antigen-presenting cells in invoking cellular responses to 21OH and linked their findings to the high-risk HLA genotype for Addison’s disease, DRB1*0301-DQ2/DRB1*0404-DQ8 (71).

Two immunodominant peptides have been found to be recognized by CD8+ T cells. Rottembourg et al. identified circulating T cells specific for a dominant 21-OH peptide in a significant proportion of HLA-B8+ patients. They further identified these responses to target the HLAB8-restricted octameric sequence EPLARLEL (position 21OH_431-438_), and most of the responding patients carried the HLA-B*0801 allele (75). A comprehensive peptide mapping was introduced by Dawoodji and colleagues using 18mer overlapping synthetic peptides spanning the entire 21-OH protein. They demonstrated that T cells from PAI individuals, unlike healthy controls, responded to the pool of 21-OH peptides. The responses were dominated by MHC class I restricted CD8+ T cells and focused on immunodominant regions on 21-OH, specifically an HLA-A2-restricted epitope (LLNATIAEV, position 21OH_342-350_), and demonstrated the ability of a 21OH_342-350_-specific CD8+ T cell clone to lyse LLNATIAEV-pulsed target cells by granzyme B release (76). Following up on these results, Hellesen et al. confirmed a higher frequency of HLA-A2 restricted LLNATIAEV specific cells in patients with PAI than in controls. These cells could also be expanded *in vitro* in an antigen specific manner and displayed a robust antigen specific IFN-γ production. In contrast, only negligible frequencies of EPLARLEL-specific T cells were detected in both patients and controls with limited IFN-γ response. However, significant IFN-γ production was observed in response to a longer peptide encompassing EPLARLEL, 21OH_430-447_, suggesting alternative dominant epitopes. Accordingly, we discovered that the slightly offset ARLELFVVL (21OH_434-442_) peptide is a novel dominant epitope restricted by HLA-C7 and not by HLA-B8 as initially postulated (77).

## 6 Suggested clinical workup, early identification, and potential targeted treatment of autoimmune PAI

### 6.1 Identifying people at risk for autoimmune diseases

Evaluation of genetic risk factors and predictive autoantibodies are merely signs of an increased risk of developing disease. For PAI, there is currently no therapy except replacement of the missing corticosteroids, and curing therapeutic solutions in the future would rely on early identification of persons at risk of developing disease. Population screening has been done for several years, exemplified by the screening of newborns for the type 1 diabetes (T1D) genetic risk factor HLA-DQB1 in Finland as part of the Finnish Diabetes Prediction and Prevention Project (DIPP) (78). Here they follow newborn children every 3-6 months and had in 2016, after a decade of implementing the program, numerous examples of maturation and a healthy outcome, as well as cases reflecting the progression toward T1D (79). HLA-DQB1 is also a risk factor in PAI, celiac disease and autoimmune thyroid disease. This is also reflected by presence of additional autoimmune disease and disease-specific autoantibodies when T1D is diagnosed, and 1% were found to be 21OH Abs positive among a cohort of 491 children diagnosed with T1D (80). Indeed, a study on >22 million inhabitants of the United Kingdom recently reported that PAI is the autoimmune disorder which is most often seen together with additional autoimmune manifestations (10). This suggests that a broader autoantigen-screening might be beneficial for these patients, with the potential to identify isolated cases of other autoimmune diseases than T1D. Combined with PRS for the different diseases, this might pose an effective way to identify people at risk. Notably, it will be an ethical challenge to communicate results of a hypothesizing risk that spans birth through to adulthood is difficult. Further, the progression from detectable autoantibodies to disease will be highly variable, and demanding for the health care system, the patients, and their families. For T1D, the FDA recently approved teplizumab as the first disease-modifying therapy in type 1 diabetes. Teplizumab is a humanized anti-CD3 monoclonal antibody found to delay the onset of stage 3 type 1 diabetes and is approved for use in adults and children aged 8 years and older in people with multiple autoantibodies and dysglycaemia (stage 2 type 1 diabetes). This gives good reasons for identifying people at risk for early follow-up and gives hope that targeted therapy of organ-specific autoimmune diseases is a future possibility also for other diseases than T1D.

### 6.2 Suggested clinical work-up and potential for targeted treatment

The last decades have provided new insight into the underlying genetic risk factors and the immune cells that contribute to adrenal tissue destruction. Environmental factors are difficult to determine in rare diseases, but involvement of viruses has been suggested to play a part (81). Still, PAI patients are diagnosed when most of the adrenal cortex is destroyed, representing the end stage of an autoimmune inflammation taking place over months and years, and several patients are still diagnosed during Addisonian crisis where the outcome might be fatal.

Interestingly, it was recently found that as much as 30% of all PAI patients have some residual hormone-producing capacity (82), which could represent a window of opportunity to rescue adrenal function, but there are still several hurdles to overcome to provide early diagnosis and targeted treatment of PAI. Although some information has been gathered on the natural course of the autoimmune destruction by observing decline in hormone level, details on how the autoimmune reactivity against the adrenal is triggered and evolves are completely unknown and this information is needed for intervention at an early stage to be successful. We also lack in-depth information about the immune system in patients with PAI. Mapping the pathogenic pathways can point to which drugs that could be effective in halting the autoimmune reaction. Identifying the exact epitopes in the autoantigens associated with adrenalitis can help develop peptide or mRNA vaccines or chimeric antigen receptor T regulatory cells (CAR Tregs) to induce immune tolerance.

**Figure 5:**
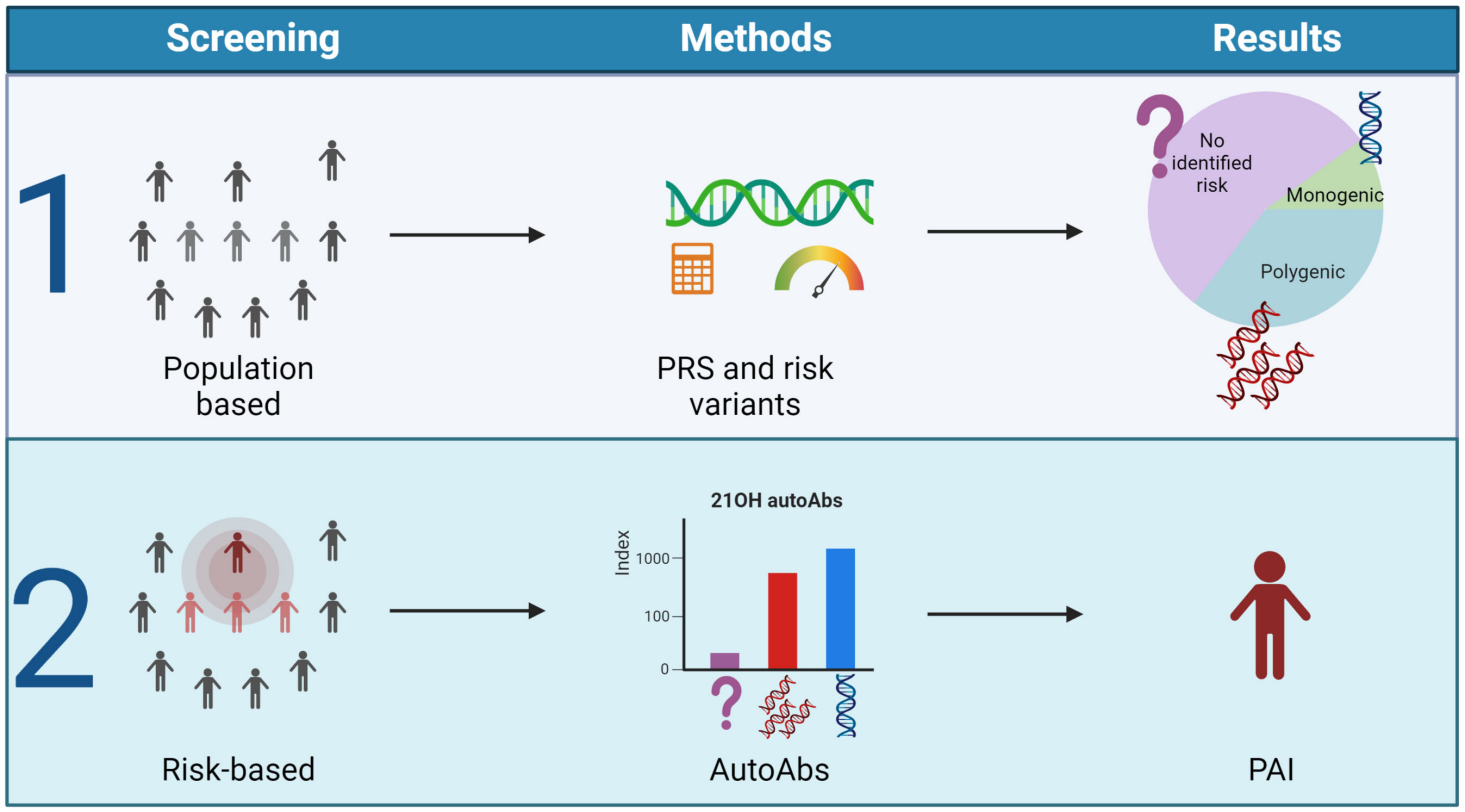
Outlined screening possibilities for PAI. The figure suggests two different strategies for early identification of PAI. Population-based screening dependent on polygenic risk scores and genetic screening of risk variants (1) or 21-OH autoantibody measurements in high-risk groups either identified by genetic screens or family history (2). Figure created in BioRender.com.

To conclude, the underlying genetic landscape in PAI is emerging, a prerequisite for future genetic population screens. These will be powerful tools together with autoantibodies targeting 21OH to identify patients earlier before the adrenal tissue is destroyed (**Figure 5**). This will be a crucial step to enable targeted therapy for this disease.

## Author contributions

AW: Conceptualization, Writing – original draft, Writing – review & editing. IK: Conceptualization, Writing – original draft, Writing – review & editing. BO: Conceptualization, Writing – original draft, Writing – review & editing.
